# Background Coloration of Squamous Epithelium in Esophago-Pharyngeal Squamous Cell Carcinoma: What Causes the Color Change?

**DOI:** 10.1371/journal.pone.0085553

**Published:** 2014-01-28

**Authors:** Hitomi Minami, Hajime Isomoto, Toshiyuki Nakayama, Tomayoshi Hayashi, Naoyuki Yamaguchi, Kayoko Matsushima, Yuko Akazawa, Ken Ohnita, Fuminao Takeshima, Haruhiro Inoue, Kazuhiko Nakao

**Affiliations:** 1 Department of Gastroenterology and Hepatology, Nagasaki University Hospital, Nagasaki, Japan; 2 Department of Pathology, Nagasaki University Hospital, Nagasaki, Japan; 3 Digestive Disease Center, Showa University Northern Yokohama Hospital, Yokohama, Kanagawa, Japan; National Cancer Institute, National Institutes of Health, United States of America

## Abstract

**Objectives:**

This study aims to clarify the cause of background coloration in the epithelia between each dilated intra papillary capillary loop in esophago-pharyngeal squamous cell carcinoma.

**Design:**

This is a single center retrospective study including 124 patients with 160 lesions who underwent esophagogastroduodenoscopy in Nagasaki University Hospital from September 2007 to March 2012; a detailed comparison between endoscopic images and pathology was performed. Immunohistological assessment using anti-human hemoglobin antibody (anti-Hb Ab) was performed to verify the presence of hemoglobin (Hb) component in the cancer cells. Real-time polymerase chain reaction (RT-PCR) and in situ hybridization (ISH) on Hb-β mRNA were performed to assess the production of Hb component within the cancer cells.

**Results:**

A strong positivity for anti-Hb Ab was observed in the squamous cell carcinoma area, whereas non-cancerous mucosa showed no immunopositivity for Hb. The concordance rate between anti-Hb Ab immunoreactivity and the presence of BC was as high as 80.9%. The amount of Hb-β mRNA expression was three times higher in cancer tissues compared with the surrounding non-cancerous mucosa. ISH images showed that the expression exclusively occurred in cancer cells, indicating that Hb is probably produced within cancer cells.

**Conclusions:**

The background coloration observed is partly due to an extravascular component of Hb. RT-PCR and ISH analyses indicate that Hb is produced within cancer cells.

## Introduction

Recently, endoscopic mucosal resection (EMR) and endoscopic submucosal dissection (ESD) have become the tools of choice for treating early gastrointestinal cancers. It has become essential for endoscopists to detect cancers when they are still at an endoscopically resectable stage. However, differentiating malignancy from benign lesions such as inflammatory change and low-grade intraepithelial neoplasm (LGIN) is extremely difficult even for experienced endoscopists. With the assistance of a narrow band imaging (NBI) system, magnifying endoscopy enables the clear visualization of fine mucosal architecture and microvasculature [1], [2]. Inoue et al. reported the significance of intra-capillary papillary loop (IPCL) change including irregular dilation and caliber changes in diagnosing esophagopharyngeal squamous cell carcinoma (SCC) [3] [4] [5]. IPCL alteration is one of the earliest changes observed in superficial SCC. More recently, we reported the significance of the brownish color change in the squamous epithelia between each IPCL via NBI-based observation, termed as background coloration (BC), as an additional endoscopic finding to discriminate early SCC from benign lesions in the esophagopharyngeal region [6]. As we mentioned in the report, the accuracy of diagnosing malignant tumors from benign ones was more than 87.3%. Furthermore, the sensitivity, specificity, positive predictive value, and negative predictive value were reported to be 91.9%, 76.7%, 90.1%, and 80.2%, respectively [6] [7].

The cause of BC is still unclear, but this phenomenon may be derived from hemoglobin (Hb) components themselves, as identified by NBI. This study evaluates the association of BC with Hb protein and mRNA expression via immunohistochemistry using anti-human Hb antibody (anti-Hb Ab), along with state-of-art techniques including in situ hybridization (ISH) and real-time polymerase chain reaction (RT-PCR) [8].

## Materials and Methods

### 1. Patients

Between September 2007 and March 2012, 124 patients with 160 esophago-pharyngeal lesions who underwent endoscopy were enrolled in this study; a detailed comparison between endoscopic images and pathology was performed for all lesions. For immunohistological evaluation using anti-Hb Ab, 43 consecutive patients with 47 lesions, who underwent endoscopic resection between February 2010 and July 2011, were enrolled.

The study was approved by the Nagasaki University Hospital Ethics Committee. All samples were obtained with the written informed consent of the patients prior to their inclusion, in accordance with the Helsinki Declaration.

### 2. Endoscopic observation

A GIF-H260Z (Olympus medical systems Co., Tokyo, Japan) comprising a high-resolution white-light video endoscope equipped with the NBI system was used. The NBI system has a high-resolution mode in which the white light image is composed of sequential images taken through red, green, and blue (RGB) bandpass filters. In the NBI mode, the central wavelengths of the RGB filters [445 nm (blue), 540 nm (green), and 620 nm (red)] are narrowed to 415 nm (blue) and 540 nm (green) [1]. Using the wavelength dependence of the light penetration depth into the mucosa and the Hb absorption characteristics in the surface layer, NBI technology allows the mucosal surface layer to be displayed in high contrast and enhances Hb-rich areas such as blood vessels [2].

Patients underwent upper endoscopy under conscious sedation with intravenous pethidine hydrochloride (35–70 mg, Mitsubishi Tanabe Pharmaceutical Co., Osaka, Japan) supplemented with diazepam (5–10 mg, Takeda Pharmaceutical Co., Osaka, Japan). In order to suppress esophageal peristalsis, either scopolamine butyl bromide (20 mg, Boehringer Ingelheim GmbH, Ingelheim, Germany) or glucagon (1–2 mg, Novo Nordisk, Bagsværd, Denmark) were also administered intravenously.

### 3. Evaluation of brownish coloration (BC)

**Figure 1:**
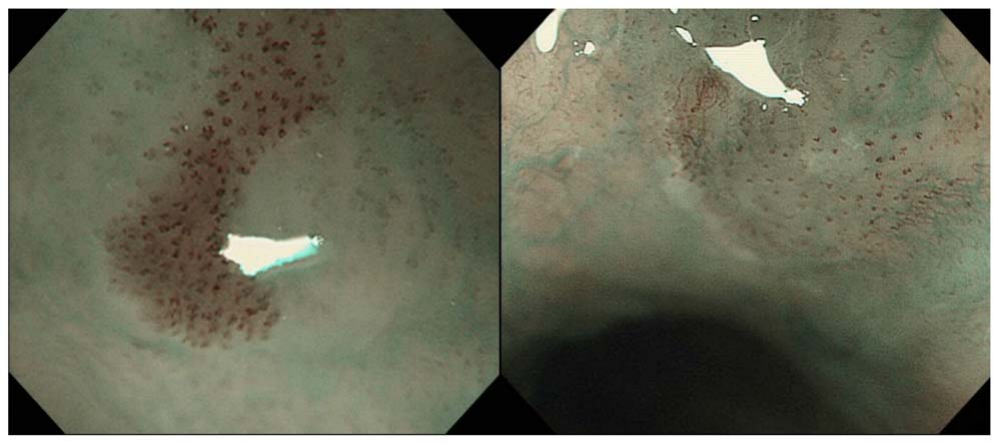
a. Endoscopic image using narrow band imaging (NBI) of the case that was positive for BC. Brownish color change is observed in the epithelia between each irregularly dilated IPCL. b. Negative for BC. The color of the epithelia between each IPCL is identical to surrounding mucosa.

After a brownish area (BA) was observed with NBI, the lesion was observed with NBI magnification to evaluate the presence of BC. If a distinct color change was observed in the area between IPCLs, the lesion was regarded as positive for BC (Figure 1a). If no color change was observed in the area, the lesion was regarded as negative for BC (Figure 1b). For larger lesions with heterogeneous BC findings, the lesion was categorized into the BC positive group when BC-positivity was clearly observed at least in one part of the lesion.

### 4. Pathological evaluation

Each specimen resected by EMR/ESD was fixed in 10% formalin and embedded in paraffin wax. The tissue specimens were cut at a thickness of 4 µm, and all sections were routinely evaluated for pathological diagnosis. Biopsy specimens collected by endoscopists in suspicion of or to rule out SCC were also used to examine diagnostic histological findings. All specimens were evaluated by experienced gastrointestinal pathologists. Histology was assessed according to the 10^th^ edition of the Japanese Classification of Esophageal Cancer (The Japan Esophageal Society) as LGIN, high-grade intraepithelial neoplasia (HGIN), or SCC. Intraepithelial neoplasia is defined as “non-invasive cytological or architectural alterations that may lead to the development of invasive carcinoma”. HGIN is associated with a high risk of progression to invasive carcinoma. In HGIN, the abnormalities including histological features and cytological abnormalities involve the upper half, and cytological alterations are greater than those in LGIN. Sometimes it is extremely difficult to discriminate between HGIN and carcinoma in situ (CIS) or non-invasive carcinoma; therefore, HGIN and CIS are classified into the same category as T1a-EP carcinoma.

### 5. Immunohistochemistry

Immunostaining was applied to the biopsied or EMR/ESD-excised specimens in the 47 consecutive lesions. The presence of Hb in human esophageal mucosa and human SCC was assessed by immunohistochemistry. Formalin-fixed and paraffin-embedded specimens were cut into 4-µm thick sections, deparaffinized, and pre-incubated with normal bovine serum to prevent non-specific binding. The sections were incubated for six hours at 4°C with the primary polyclonal antibody to human Hb (1 µg/mL; Bethyl Laboratories, Inc., Montgomery, TX, USA) and then with a labeled donkey anti-goat IgG antibody (Alexa Flour 555, 0.4 µg/mL; Invitrogen, Carlsbad, CA, USA). Negative controls involved replacing the primary antibody with non-immunized bovine serum and the human respiratory tissue served as the positive control [9]. Cell nuclei were stained with 4′,6-diamidino-2-phenylindole (DAPI, Thermo Fisher Scientific, Waltham, MA, USA).

**Figure 2:**
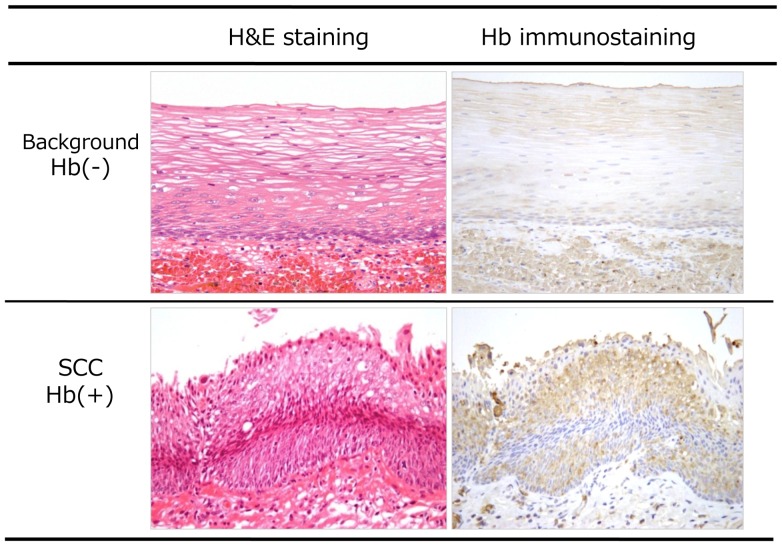
Comparison of immunohistological images using anti-Hb Ab. Immunostaining image showed intense positivity as red blood cells in the cancer area. Non-cancerous mucosa revealed to be negative for anti-Hb Ab.

Anti-Hb immunostaining intensity was classified as follows: (+) similar to intravascular red blood cells; (−) identical to surrounding non-neoplastic epithelium (Figure 2). Immunostaining using anti-p53 and anti-ki-67 antibodies was also performed to determine the pathology of the lesion.

### 6. RT-PCR and in situ hybridization

#### RNA extraction and reverse transcription

During endoscopy, two biopsy specimens were obtained from each neoplastic and non-neoplastic lesions and snap-frozen for relative mRNA quantification of Hb-β (HBB). Total RNA was isolated from the samples using the mirVana miRNA Isolation Kit (Ambion). Each RNA concentration was determined by spectrophotometry. The complementary DNA synthesis was performed using 6 µL of 10× RT Random Primer, 6 µL of 10× RT buffer, 2.4 µL of 25× dNTP Mix, 3 µL of RNase inhibitor, 3 µL of MultiScribe Reverse Transcriptase (Applied Biosystems), and 9.6 µL nuclease-free water (Ambion); 400 ng of total RNA was used in each reaction. The reactions were carried out at 37°C for two hours followed by five minutes incubation at 85°C. The cDNA samples were stored at −20°C until the real-time RT-PCR assays.

#### Real-time RT-PCR for HBB

The expressions of HBB and glyceraldehyde 3-phosphate dehydrogenase (GAPDH) were quantified using LightCycler 480 II Instrument (Roche), according to the manufacturer's protocol. The reaction for the real-time PCR using TaqMan detection consisted of 25 µL of a Master Mix II (Applied Biosystems); 2.5 µL of a mixture containing 20× TaqMan Gene Expression Assay Mix (Applied Biosystems); 22.5 µL cDNA (HBB) and 2.5 µL cDNA (GAPDH); and 20 µL water (GAPDH). The reactions were performed in a LightCycler 480 Multiwell Plate 96 (Roche). All samples were incubated at 95°C for 10 min and then cycled at 95°C for 15 sec and 60°C for one minute for 40 cycles. Controls with no template cDNA were also analyzed in each assay. The mRNA expression levels were quantified using the crossing point value. Relative gene expression levels were obtained using the two-crossing point value method. Each sample was tested in duplicate.

#### Fluorescent in situ hybridization (FISH) for HBB mRNA

RNA-FISH was performed using QuantiGene® ViewRNA ISH Tissue Assay kit, according to the manufacturer's protocols (Affymetrix). Tissue sections (4 µm) were deparaffinized, fixed in 4% phosphate-buffered paraformaldehyde, boiled in pre-treatment solution, and digested with proteinase K (included in kit). Sections were hybridized for three hours at 40°C with a probe designed against the human HBB and ubiquitin-C (UBC) (Affymetrix). Hybridized probes were then amplified using PreAmp and Amp molecules. Multiple Label Probe oligonucleotides conjugated to alkaline phosphatase (LP-AP, included in kit) were then added and FastRed (for HBB) or FastBlue (for UBC) substrates were used to produce fluorescent signals. Nuclei were then counterstained with DAPI. As for fluorescent imaging and processing, a series of high-resolution monochromatic images were captured using an automated microscope platform equipped with 60× objective magnification (PM-2000TM; HistoRx). Fluorescent filters (DAPI, Cy3, and Cy5) were used to detect dual-color RNA-FISH signals within each section. Images were depicted by pseudo-color merging using the image processing software ImageJ (National Institutes of Health, USA). HBB signals (Cy3) were colored red and UBC signals (Cy5) were green; nuclei were colored blue (DAPI).

### 7. Statistical analysis

Statistical analyses were performed using the χ^2^ test and Student's *t*-test. A *P*-value<0.05 was accepted as statistically significant. Data were expressed as mean ± standard deviation (SD).

## Results

### 1. Background coloration; BC

Details of the lesions are shown in Table 1. One hundred and thirty seven out of the 160 lesions showed BC positivity. Only four (2.9%) lesions were shown to have benign pathology in the BC positive lesions. On the other hand, 43.5% were diagnosed as a benign change in the BC negative group. The sensitivity, specificity, and overall accuracy of BC in differentiating malignancy from benign pathology were 91.1%, 71.4%, and 89.4%, respectively. Of the 14 non-malignant lesions, 10 (71.4%) were negative for BC. Furthermore, 133 (91.1%) lesions of HGIN/SCC were positive for BC and 97.5% of invasive carcinoma (deeper than LPM) was positive for BC.

**Table 1:** Correlation between BC positivity and the pathology.

Background coloration		Pathology		
BC(+)	137	Inf/LGIN	4	[2.9%]
		HGIN/SCC	133	[97.1%]
BC(−)	23	Inf/LGIN	10	[43.5%]
		HGIN/SCC	13	[56.5%]

97.1% of BC positive lesions were diagnosed as malignant. Overall accuracy of differentiating malignancy from benign pathology was 89.6%.

### 2. Immunohistochemistry

**Figure 3:**
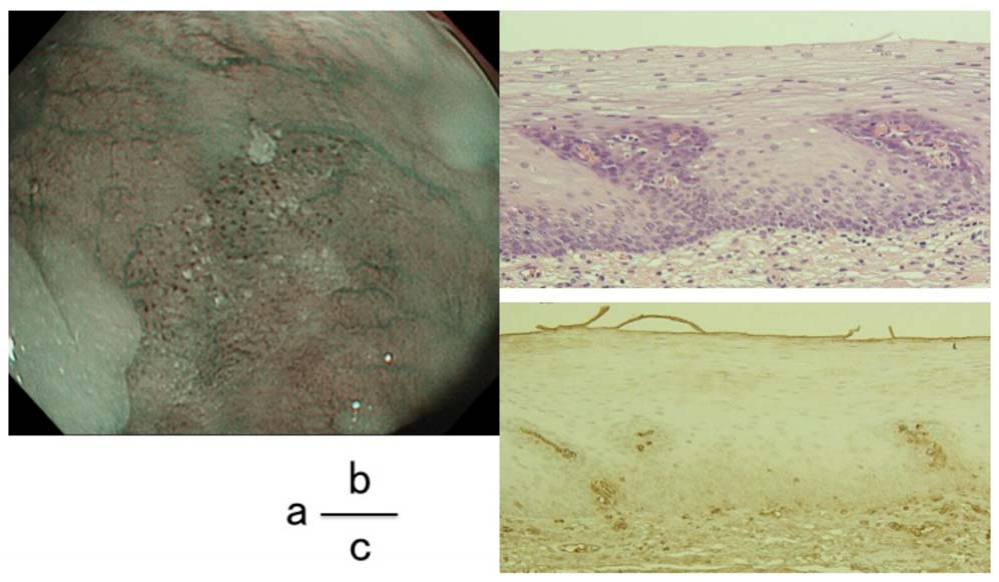
BC-negative case. a. The color of the epithelia between each dilated IPCL was identical to surrounding mucosa. b. H&E staining histologically confirmed the lesion as intraepithelial neoplasia. c. Immunohistology using anti-Hb Ab showed that the lesion was negative for Hb.

**Figure 4:**
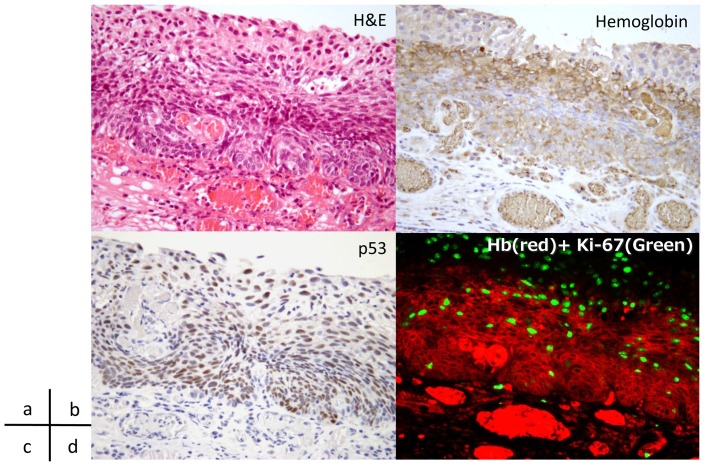
Immunohistochemical images of a case of SCC. a, b. Hematoxylin and eosin staining of cancer lesion. Carcinoma cells are observed in lamina propria mucosae. Cancer area was positive for hemoglobin immunostaining. c. The extent of cancer area was confirmed using p53 immunostaining. d. Immunofuolescent multistaining image showed that the immunopositivity is localized in the cancer cells.

Table 2 shows the results of the immunohistological evaluation. Among the 47 lesions examined, 36 were positive for BC and all the BC positive lesions were shown to be positive for anti-Hb Ab immunostaining. However, nine of 11 lesions that were negative for BC showed anti-Hb Ab negativity. The concordance rate between BC positivity and anti-Hb Ab immunoreactivity was higher than 80.9%. Two lesions that were negative for anti-Hb immunostaining were also negative for BC (Figure 3 a–c). Only 2.2% of the anti-Hb negative group was pathologically negative for malignancy and the two lesions that were anti-Hb negative were also negative for malignancy. Immunohistochemical images revealed that red blood cells in capillaries or vessels were clearly positive for Hb immunostaining as well as SCC cells (Figure 4 a, b). A clear boundary was observed between the cancerous and non-cancerous areas; the anti-Hb Ab positive area was perfectly matched to cancer area, which was confirmed as carcinoma using p53 immunostaining (Figure 4c). On the other hand, the surrounding non-cancerous epithelia showed nominal or negative staining for Hb. Moreover, immunopositivity for Hb was exclusively localized in the cancer cells outside of nuclei that were stained with DAPI (Figure 4d). We also counted the number of Hb-positive cells in 100 cells within the cancer area. The mean positive rate of Hb immunostaining was 83.8% (range; 23–100, median 87).

**Table 2:** Immunohistological results.

Hb BC	(+)	(−)	total
(+)	36	9	45
(−)	0	2	2
total	36	11	(n = 47)

All 36 lesions that were positive for Hb were also positive for Hb immunostaining. Concordance rate of Hb immunopositivity and BC positivity was high as 80.9% (36+2/47).

### 3. RT-PCR/ISH

**Figure 5:**
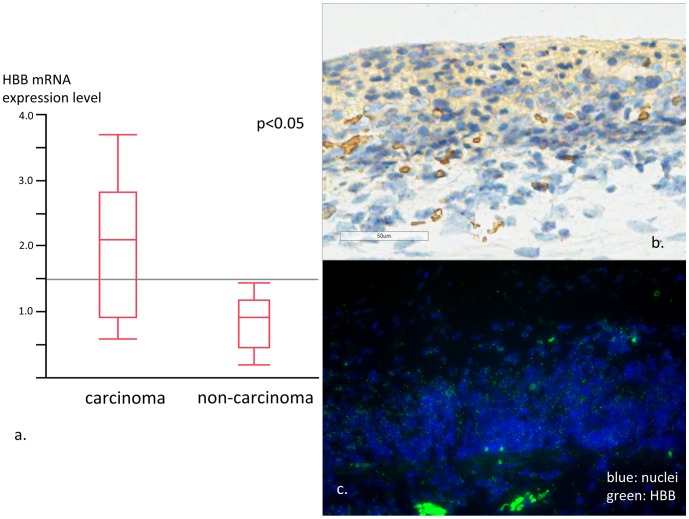
Results of RT-PCR and in situ hybridization. a. Expression level of HBB was significantly higher in carcinoma tissue compare to non-cancerous surrounding tissue. The amount of expression varied highly in cancer tissue. On the other hand, the expression level contributed to relatively narrow range in non-cancerous tissue. b. ISH image from cancer area. HBB mRNA expression (red) was observed in the cancer area. c. ISH fluorescent image from cancer area showed strong positivity for HBB mRNA expression (green).

The HBB mRNA RT-PCR and ISH results are shown in Figure 5 a–c, respectively. RT-PCR results revealed that HBB mRNA expression level was three times higher in the cancerous area than in the surrounding non-cancerous mucosa (*P*<0.05). ISH images showed the HBB mRNA expression was localized mostly within the cytoplasm of cancer cells while only a small amount of expression was observed in non-cancerous tissues. These results indicate that Hb may be produced by cancer cells.

## Discussion

Conventional endoscopic diagnostic skills such as IPCL pattern classification [4] via NBI-based endoscopic observation and pink coloration [10], [11] with standard observation have been reported useful in discriminating between early SCCs of the esophagopharyngeal region. However, dilation and tortuous changes of IPCL could be observed in benign lesions including inflammatory changes and LGINs. Precise differentiation of cancer from other benign changes is extremely difficult even for experienced endoscopists.

NBI is one of the most reliable diagnostic tools for diagnosing early gastrointestinal cancers. Finding BA is the first step in the discrimination of early esophageal SCCs, as it is commonly the area with irregularly dilated IPCLs. Furthermore, the brownish color change of epithelia between each IPCL (BC) has been reported as a cause of the BA [6], [7]. We have reported the significant association of esophagopharyngeal SCC with BC [7].

NBI technology allows the mucosal surface layer to be displayed in high contrast and enhances Hb-rich areas including blood vessels as BC [2]. However, there is no reasonable explanation as to why the neoplastic esophageal epithelia could be homogeneously positive for the specific wavelength of Hb. Here we conducted immunohistochemical staining with anti-Hb Ab to explore the underlying mechanisms of BC. The immunofluorescent staining images using anti-Hb Ab revealed that the SCC cells showed strong immunoreactivity as observed in the intra-vessel reticulocytes. Moreover, Hb expression was localized in the cytoplasm but not in the nuclei of carcinoma cells.

Roesch-Ely et al. reported the expression of Hb-α and HBB in SCCs of the head and neck region [12]. Other neurological immunochemistry reports have suggested the possibility of Hb existing in normal physiology outside the blood cells as indicated by an overexpression of Hb-α and HBB chains in dopaminergic cell lineages [13]. Given that the immunohistochemistry employed here only detects HBB at the protein level, positive staining does not exclude the possibility of Hb uptake by cancer cells from the surrounding tissues. We therefore performed additional experiments including RT-PCR and ISH to detect HBB at the mRNA level.

The results of RT-PCR on mRNA of HBB supported the possibility that Hb could be produced in esophageal SCCs. Furthermore, ISH images revealed that mRNA expression was mainly observed in the cancer cells. The expression level varied widely compared with in surrounding non-cancerous mucosa according to the RT-PCR data. Taken together, these results established that esophageal cancer cells themselves are capable of synthesizing Hb.

The degree of BC change was observed to be heterogeneous even in the same lesion; RT-PCR results may explain the reason for this discrepancy. Further studies are required to verify the correlation between the quantity of color change and the amount of Hb expression. Kanzaki et al. reported that thinning of the keratinous layer by neoplastic cell proliferation is responsible for the BC [14]. This, along with Hb expression in cancer cells, might explain the unevenness of the brownish color change. On the other hand, massive submucosally invasive cancers, which lack an appropriate keratinous layer, sometimes appear to be negative for BC; this is contrary to the correlation between the thickness of the keratinous layer and color change. Further studies are mandatory to determine the exact cause of BC.

There are few studies on the production of Hb by SCC cells. Liu et al. studied the Hb expression of hepatocytes in non-alcoholic steatohepatitis (NASH) and reported that suppression of oxidative stress by Hb could be a mechanism for hepatocyte protection from oxidative damage in NASH [15]. In addition, malignant tumors are known to reveal higher ratios of oxygen uptake than normal tissues. Ogane et al. reported that the expression of hypoxia inducible factor-1 alpha (HIF-1α) was significantly higher in cases with cancer metastasis in their 96 surgically resected T1b esophageal SCC cases [16]. It is possible that Hb expression is related to oxidative stress or a low oxygen environment. Other studies have reported Hb expression in non-erythroid cells such as alveolar epithelial cells [17] and renal mesangial cells [18]. The role of Hb might be related to gas change, nitrogen oxide metabolism, and protection against oxidative and nitrosative stress. Further investigation with a larger cohort is required to assess the causes and mechanisms of Hb expression in SCC cells.

## Conclusions

BC is a useful additional finding to discriminate between neoplastic lesions in the esophagopharyngeal region. The extravascular Hb component can be considered as one of the causes for the observed color change. RT-PCR and ISH results indicate that Hb is produced in cancer cells; however, further investigations are required to clarify the underlying mechanisms of BC.
